# Approaches to the Mechanical Properties of Threaded Studs Welded to RHS Columns

**DOI:** 10.3390/ma14061429

**Published:** 2021-03-15

**Authors:** Ismael García, Miguel A. Serrano, Carlos López-Colina, Jesús M. Suárez, Fernando L. Gayarre

**Affiliations:** Department of Construction and Manufacturing Engineering, University of Oviedo, 33203 Gijón, Spain; garciaismael@uniovi.es (I.G.); serrano@uniovi.es (M.A.S.); suarezg@uniovi.es (J.M.S.); gayarre@uniovi.es (F.L.G.)

**Keywords:** welded studs, bolted connections, small punch test, mechanical characterization

## Abstract

The use of Rectangular Hollow Sections (RHS) as columns in steel construction includes important advantages like higher mechanical strength and fire resistance. However, the practical demountable bolted joints between beams and columns are not easy to execute, due to impossibility of access to the inner part of the tube. The use of threaded studs welded to the face of the tube and bolted to the beam by means of angle cleats is one of the cheaper and most efficient solutions to obtain beam–column joints with a semi-rigid behavior, as is usually sought in building structures. Nevertheless, it is important to point out that the stud-diameter and the stud-class selection may affect the mechanical properties of the welded parts of the joint. In this paper, 8MnSi7 (with a commercial designation K800) and 4.8 threaded studs were welded to RHS steel tubes and mechanical properties on the weld, the Heat Affected Zones (HAZ), and the base metal were obtained in two different ways: through a correlation with the Vickers hardness and by means of the Small Punch Test (SPT). A study of the microstructure and tensile tests on the threaded studs and in the columns was also carried out. The research involved different types of stud qualities, tube wall thicknesses, and stud diameters. The work presented in this paper proved that in most cases, the welded joint between these studs and the RHS steel tubes present a reasonable static behavior that fulfils the requirements for the beam–column joints under static loading.

## 1. Introduction

Steel building construction has traditionally chosen open steel sections such as I or H-profiles as beams and columns. However, the use of structural hollow sections as columns shows advantages like their mechanical strength, fire resistance, maintenance costs, aesthetics, and the possibility to create composite columns and making better use of space. Most of the beam–column joints between Rectangular Hollow Sections (RHS) columns and open section beams are welded because the use of bolted joints in hollow sections is more difficult than in open profiles due to the impossibility to access to the inner part of the tube. Nevertheless, the benefits of using demountable steel connections lead some authors to propose solutions consisting of bolted joints in tubular columns. They can require intermediate pieces welded to the tube as the reverse channel connections using standard bolts [1] or expensive blind bolts [2,3,4]. Bolted joints with threaded studs welded to the front face of the hollow section column were also proposed [5,6] as a fast and efficient solution.

The mechanical properties of structural materials are parameters that have to be known for the design of structures (structural elements and joints) and for the evaluation of structural integrity. These parameters (yield stress, ultimate tensile strength, Young’s Modulus, Poisson’s ratio) are obtained by standard tensile testing [7]. However, in some cases, this type of test cannot be carried out because of the size of the components [8] (typical specimens cannot be extracted) or due to the change of these properties along the component as it happens in the Heat Affected Zones (HAZ) in a welding process [9]. In these cases, the mechanical properties have to be obtained in a different way. Two indirect methods to get the mechanical properties are through a correlation with the Vickers hardness [10,11] and through the Small Punch Test (SPT) [12].

Hardness tests determine the ability of a material to resist very localized deformations and is widely used to determine comparative hardness numbers for metals. This is because the hardness tests are simple and quick to execute. Different authors have proposed equations [10,11] to convert a given hardness number into some others of the mechanical properties of the material.

SPT was initially developed in the 1980s [13] for nuclear applications and it consists of two circular dies with a circular hole and a hemispherical punch. The small-size specimen is located between two dies, while at the same time, a punch indents the specimen perpendicularly. The applied load and the displacement of the punch are recorded, and its relations can be used to estimate the yield stress [13,14,15,16,17], the ultimate tensile strength [14,16,17,18], and the elongation [9]. The broken specimen can also be used to obtain an estimation of the fracture toughness [19,20,21,22]. Although the use of SPT has spread to other industries and a European Code of Practice [23] was published in 2006, there is currently no SPT standard covering most of the widely used applications [24].

In this paper, small punch tests were carried out on samples selected from threaded studs and the tube walls where the threaded studs were welded to obtain the mechanical properties on the welds, the heat affected areas, and the base metal. In the same places, hardness tests were done in order to compare the mechanical properties obtained with SPT and obtained through the hardness number. The research involved two different types of stud classes, two RHS wall thicknesses and two different stud diameters. Firstly, studs with different alloy elements (classes 4.8 and K800) with a metric M16 and M20 were welded to structural steel tubes with wall thicknesses of eight and ten millimeters making up six objects with different combinations. Then, for every one of the six connections, seven small specimens were prepared from different locations of the welded stud and tube wall in order to obtain their hardness and microstructure. Finally, the specimens were tested using SPT and mechanical properties such as yield stress, ultimate tensile strength, and elongation were estimated from these tests. The results were compared with those obtained from tensile tests carried out on coupons of the complete threaded studs and on the base metal from tubes. A Digital Image Correlation (DIC) system was used for a better accuracy of these tensile tests.

## 2. Materials and Methods

### 2.1. Welding Process

Three types of studs were welded to any of the two different structural hollow sections. So, six combinations tube/studs were prepared. Metrics M16 and M20 and strength classes of 4.8 and K800 (commercial name for 8MnSi7 steel studs) were employed. The studs were welded to two different thicknesses of the tube, according to Table 1. The thicknesses of the structural tube were chosen according to the ISO standard [25], taking thicknesses higher than the recommended minimum values of 4 mm for M16 and 5 mm for M20. The tubes were made of S355J2H.

The 8 mm thickness tube consisted of a Rectangular Hollow Section (RHS) of a S355J2H steel, while the 10 mm thickness tubes consisted of a Square Hollow Section (SHS) with the same steel grade. The tubes were manufactured by cold-forming by CONDESA. The chemical composition of a typical 4.8 steel grade stud [26], 8MnSi7 (K800) stud [27], and S355 J2H tube [28] are shown in Table 2. The chemical composition of the tubes and studs used in this study are shown in Table 3. Mechanical parameters of the S355J2H are presented in [28]. Although the general recommendation for a good weldability is that the carbon equivalent should be less than 0.35% [29], a stud carbon content lower than 0.2% is also recommended [26], whereas the critical metal parameter for welding cracking (PCM) must be lower than 0.45%. High carbon content leads to the formation of hardening cracks.

**Figure 1:**
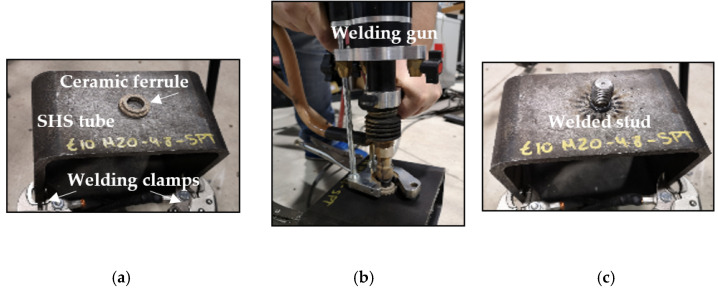
Welding process of the studs with INOTOP 1704 (Köco). (**a**) Positioning of ceramic ferrule; (**b**) welding gun before the arc; (**c**) stud welded to the tube.

The process of welding was carried out with the stud welding equipment INOTOP 1704 by Köco, Figure 1b. After cleaning the surface of the tube, a ceramic ferrule was positioned onto the workpiece (Figure 1a) and threaded studs were inserted into the chuck of the stud welding gun. The welding parameters adjusted in the gun for the M16 studs were: a current of 1000 A applied for 500 ms with a lift of 1.7 mm between the stud and the tube. For the M20 studs: 1150 A for 650 ms with a 2.2 mm lift. Then, the stud welding gun was positioned perpendicularly to the structural tube (Figure 1b). Two rods in the welding gun allow this. Finally, by clicking on the trigger the main arc between the tip of the stud and the workpiece turned on and the stud and the tube joined tightly (Figure 1c). The selection of suitable welding parameters is essential to avoid defects in the welds. Figure 1 shows the welding process and the final stud/tube specimen obtained.

The welding process was recorded by means of the thermographic camera Flir Systems AB SE-182 with a measurement temperature range of 0–360 °C. The camera was placed at a distance of 1 m from the test specimens to avoid any damage on it. The camera recorded the process of welding from the starting point to approximately 2 min after the weld was completed.

**Figure 2:**
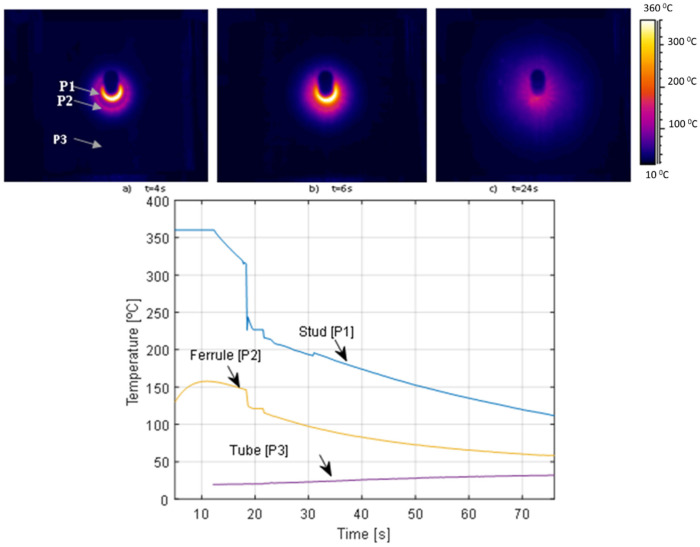
Distribution of temperatures along the tube surface: (**a**) 4 s after the arc welding, (**b**) 6 s after the arc welding, and (**c**) 24 s after the arc welding. In the graph, cooling rate of stud (P1), ceramic ferrule (P2), and tube (P3).

The analysis of temperatures recorded by the thermographic camera showed the high values of temperature around the stud. Figure 2 shows the distribution of temperatures along the tube surface of a specimen with a 16 mm diameter stud of class K800 welded to an 8-mm wall tube at different times: 4, 6, and 24 s after the welding process. Figure 2a,b show the stud with the ceramic ferrule, while in case Figure 2c the ferrule was suppressed. It must be taken into account that the stud welding gun and the ceramic ferrule do not allow to see the surface just in the moment when the arc weld takes place. The cooling rate of three reference points corresponding to stud (P1), ferrule (P2), and tube (P3); are shown in the graph in Figure 2. The graph shows some temperatures out of range of the thermographic camera (>360 °C) around the stud (reference point P1), while the rest of the tube remains at lower temperatures (reference point P3). From the graph it can be observed that point P3 increases temperature with time; which reveals the heat transfer due to the thermal conduction from the stud to the rest of the tube. The temperatures recorded suggested that no microstructural phase changes are expected in the tube in an area far away from de stud, i.e., only microstructural phase changes near the stud are expected. All the six specimens were analyzed in terms of temperature variations with the same procedure and similar results were found for the rest of the specimens.

### 2.2. Procedure of Getting the Samples

**Figure 3:**
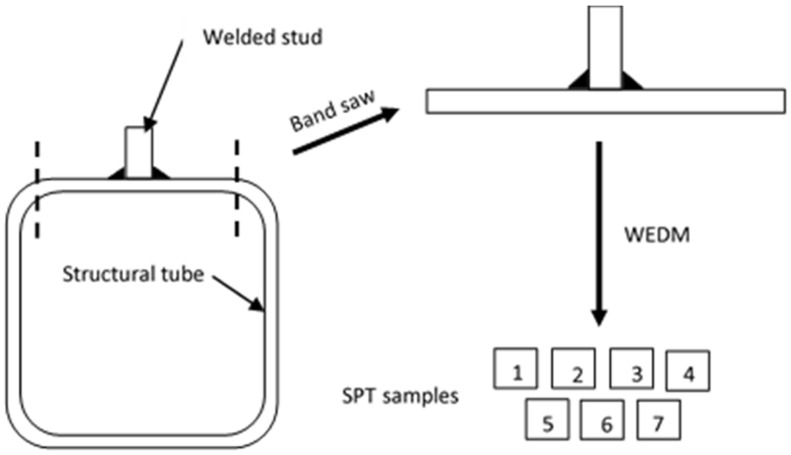
Procedure for preparing the samples through band saw and Wire Electrical Discharge Machining (WEDM).

**Figure 4:**
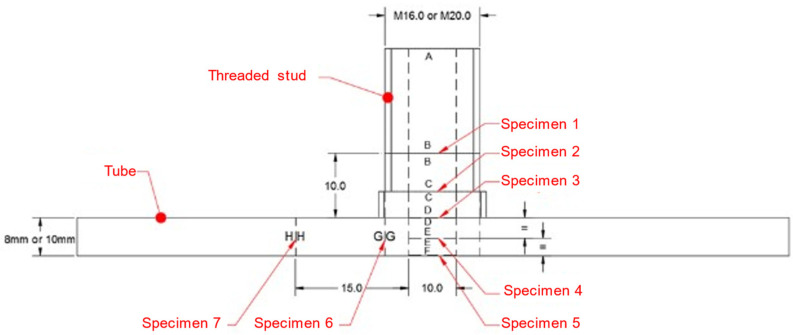
Sketch of the welded stud with the positions of the specimens (distances in mm).

Once all joints were welded, the faces of the tubes with the stud were cut with a band saw obtaining a T piece, as shown in Figure 3. After that, seven specimens of 10 × 10 × 0.5 mm were prepared by means of Wire Electrical Discharge Machining (WEDM), according to Figure 3. WEDM is a manufacturing process that allows to extract very small and thin specimens using a wire that cuts with high precision. The specific locations for the specimens are shown in Figure 4.

Then, the specimens were sanded to remove the outer oxide layer. For the sanding procedure, a die-shaped tool was used. The specimen was inserted in one of their grooves with a depth of 0.5 mm to be able to obtain the desired final thickness. Sanding was carried out in two stages using two different sandpaper grits.

Three measurements of the specimen thickness were performed in three points of the surface using a digital micrometer with a resolution of 0.001 mm. The averages of these three measurements along the thickness, are shown in Tables 7–12 for every sample. Although the specimen thickness requested to the WEDM operator was 0.5 mm, the actual thicknesses for specimens 6 and 7 was slightly lower than those for samples 1 to 5.

The seven extracted samples (from 1 to 7 showed in Figure 4) were used to small punch test, while the adjacent areas of that samples (from area B to area H showed in Figure 4) were used to carry out hardness measurements and to study the microstructure.

### 2.3. Hardness

Indentation hardness measurements were taken on parts of the weld, HAZ, and the base metal in order to evaluate the range of hardness values across the joint. Vickers hardness tests were carried out using a load of 31.12 kg. Table 4 shows the Vickers hardness obtained in zones B to H of the specimens defined in Figure 4, where B is the adjacent surface of the specimen 1, C is the adjacent surface of the specimen 2, and so on. The first column refers to the tube code formed by the thickness followed by the diameter and class of the welded stud. Therefore, the specimen code ‘T8 M16 K800′ means a class of K800 with a 16 mm diameter stud, welded to a tube of 8 mm thickness. It should be considered that the most usual notation for the Vickers hardness number has been selected here. This is the one without units but representing kgf/mm^2^ according to the physical sense of the test.

As Table 4 shows, the measured hardness values in the analyzed zones were in concordance to [26] and [30], where a range of 130 to 220 HV for 4.8 class studs are recommended. K800 studs evidenced an increase in hardness in zone D. However, hardening values are within the range proposed in [30] for class 8.8, where a hardening range between 250–320 HV for M16 studs and from 255 to 335 HV for M20 studs is recommended.

Although hardness tests are used for determining the resistance to indentation, some authors proposed equations [10,11,31,32] to relate the hardness number and the mechanical properties of the material.

#### Yield Stress and Ultimate Tensile Strength Based on HV Number

Yield stress (described as *f_y_* in this paper, like in the Eurocodes) can be obtained in MPa from HV according to the following equation by Fujita and Kuki [10]:

f_y_* = 2.736·HV − 70.5 (1)

while ultimate tensile strength (described as $f_{u}$) in this paper, like in the Eurocodes) can be obtained in MPa with the equation by the same authors [10]:

f_u_* = −160 + 4.02·HV (2)

A similar expression for the ultimate tensile strength in MPa was also proposed by Murphy and Arbtin [11]:

f_u_* = 2.5·HV + 100 (3)

The yield strength and the ultimate tensile strength estimations with the above-mentioned equations are shown in Tables 6–11.

### 2.4. Macrographs, Micrographs, and Microstructure

**Figure 5:**
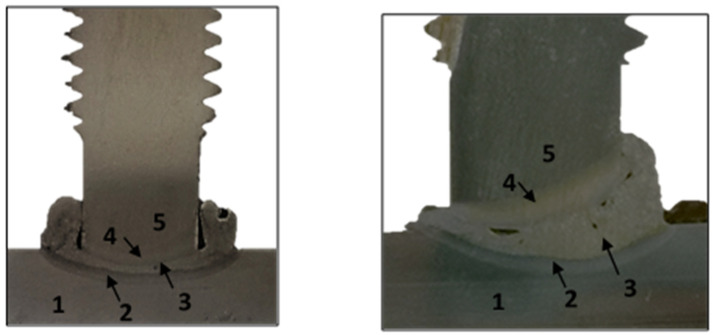
Macrograph of the T8 M20 4.8 welded stud (**left**) and T8 M20 K800 (**right**).

A visual study of the welded joint was carried out. Firstly, a pair of macrographs of the sectioned T8 M20 4.8 and T8 M20 K800 welded stud specimens are shown in Figure 5 and then, microstructural studies of the studs, HAZ and the material base were carried out. The sections were sanded using 180, 240, 400, and 800 grit sandpaper and later polished with 6- and 1-μm diamond pastes. Finally, the sections were attacked by 2% nitric acid (nital) to reveal its biphasic structure.

Five different zones are identified in the macrograph of the T8 M20 4.8 sectioned welded stud, shown in Figure 5. Zone 1 is the base material (tube wall), while zone 2 is HAZ of the tube wall and zone 3 is the fusion zone. Zone 4 is the HAZ of the stud and zone 5 is the base material (stud). From the image it is inferred that during the welding process, part of the metal finished in the corner between the stud and the tube wall. However, no fusion between this metal and the stud was observed. Furthermore, welding bead symmetry was found in every 4.8-class specimens. However, welding bead which was strengthened and raised on one side was found in T8 M20 K800 specimen Figure 5, right, which led us to think that the current distribution in the work piece was not regular, according to [25]. In that case, cracks were also found.

**Figure 6:**
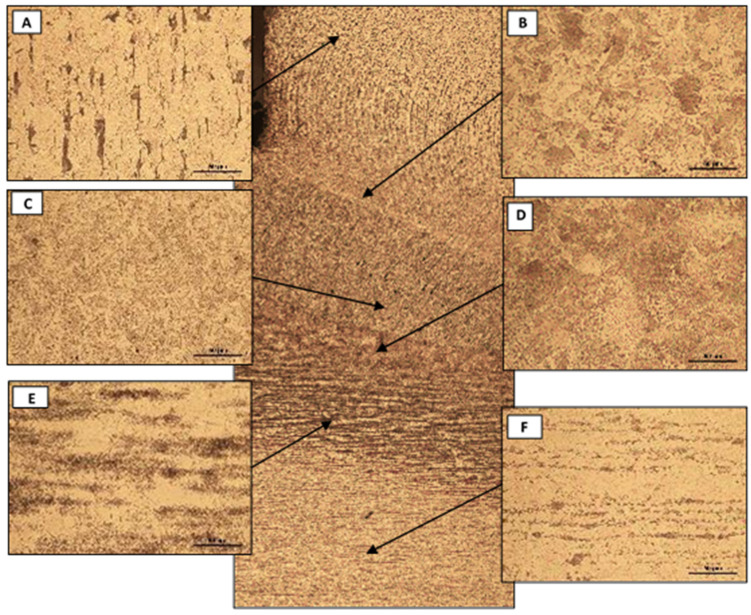
Micrographs of the T8 M20 4.8 welded stud. (**A**) unaffected base material of stud; (**B**–**D**) different ferrite and bainite microstructure; (**E**) ferrite and perlite; (**F**) unaffected base material of tube wall.

Micrographs of the different zones showed in Figure 5 were obtained. Figure 6 shows six micrographs at 500 magnification extracted from the T8 M20 4.8 welded stud. Micrographs A and F show the unaffected base metal (stud and tube wall, respectively) where a microstructure of ferrite and pearlite forming parallel alternating bands of ferrite and pearlite and a fine grain size was observed. Micrographs B, C, and D show a ferrite and bainitic microstructure with large grains. The ferrite is expressed with a Widmanstätten pattern in B and D. E shows a microstructure of ferrite and pearlite where a coalescence of pearlite was observed.

In addition to the previous study, five specimens of the T8 M20 K800 joint with a size of 10 × 10 mm and two with a 10 × 8 mm size were extracted from zones B to H, defined in Figure 4. The specimens were encapsulated in a metallographic hot mounting press to improve its handling.

**Figure 7:**
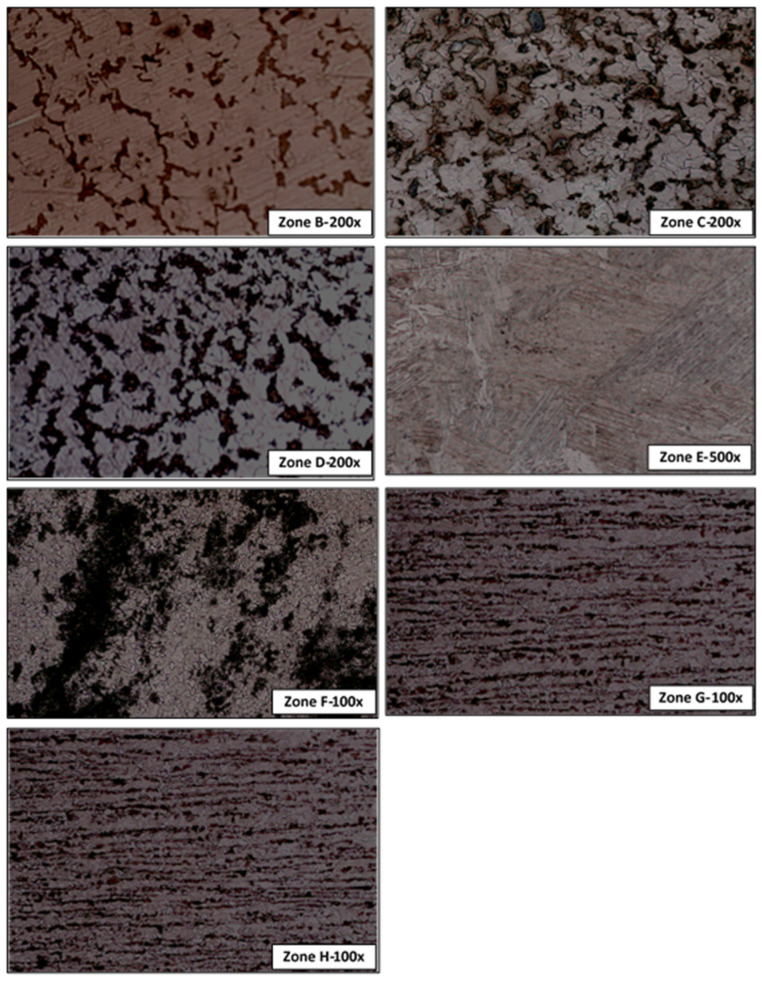
Micrographs of the different analyzed zones (T8 M20 K800).

**Figure 8:**
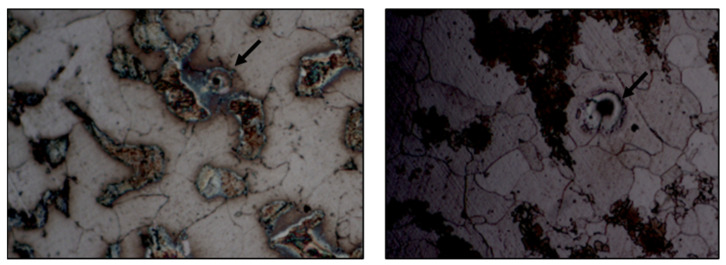
Porosity found in zones 2 (**left**, 500×) and 3 (**right**, 1000×).

The specimens were scrutinized in an optical microscope NIKON EPIPHOT 200. The micrographs obtained are shown in Figure 7. In zones B to D, the microstructure observed was formed by martensitic islands dispersed in a ferrite matrix. While in zone B the ferrite was clearly predominant, the islands were bigger in zones C and D than in zone B. Focusing the micrographs at 1000× magnification, the contour edges of martensite in zone D were more irregular than in zones B and C. Furthermore, porosities were found in zones B and C (see Figure 8).

Zone E is formed by a ferritic–bainitic structure with presence of Widmanstätten ferrite. A ferrite and pearlite microstructure with an incomplete grain recrystallization and a coalescence of pearlite was observed in zone F, while a microstructure of alternating bands of ferrite and pearlite oriented in the rolling direction was observed in zones G and H.

Some pores were found in zones 2 and 3, as it is shown in Figure 8.

### 2.5. Small Punch Tests

**Figure 9:**
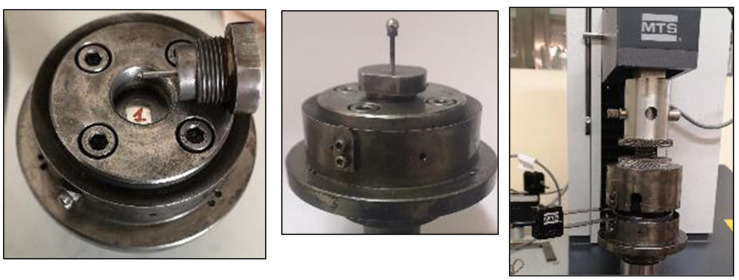
Specimen 1 placed in the SIMUMECAMAT SPT testing device (**left**). Punch rod (**middle**). SPT testing machine (**right**).

The small specimens obtained as described in Section 2.2 were placed in the lower die of the SPT testing device as shown in Figure 9, left. The SPT testing device consisted of a punch rod to which a 2.4-mm diameter steel ball was attached to the lower-end. The steel ball was lubricated to decrease the effect of friction. This punch rod was placed in an intermediate piece, which was screwed to the lower die, embedding the specimen (Figure 9 on the middle). The device was coupled to a universal MTS testing machine with a 5-kN load cell. The loading was applied by an upper die, which made contact with the punch rod by means of another ball of greater diameter located in the punch upper end. The ball prevented the transmission of bending between the machine and the punch rod, which guaranteed the axial load. During the test, the loading and displacement between the upper and lower dies were measured using an MTS COD-type extensometer. The tests were carried out under displacement control mode, at a rate of 0.2 mm/min.

As two different stud metrics, two different classes and two different tube wall thicknesses were studied, a total of six studs were welded. For each joint, seven specimens were obtained, which means 42 samples tested by SPT.

#### Yield Stress and Ultimate Tensile Strength Estimation through SPT

Yield stress ($f_{y}$) was obtained in MPa from SPT curves according to [12] by Equation (4):

(4) $$f_{y}^{**}=\alpha \frac{P_{y}}{t^{2}}$$

where α is a test constant with a value of 0.346 according to T.E Garcia et al. [12], *P_y_* is the load at the intersection between the SPT curve and a parallel line to the initial slope with an offset of *t*/10, being *t* the average thickness of the sample.

The ultimate tensile strength ($f_{u}$) in MPa can be calculated using Equation (5):

(5) $$f_{u}^{**}=\beta \frac{P_{max}}{t\cdot d}$$

where β is another test constant with a value of 0.277 according to [12], *P_max_* is the maximum value of force, *t* is the average thickness of the specimen, and *d* is the displacement at *P_max_*.

The elongation (*A*) can be obtained as:

(6) $$A^{**}\left[\%\right]=6.07\frac{d}{t}$$

### 2.6. Tensile Tests on Tubes and Studs

**Figure 10:**
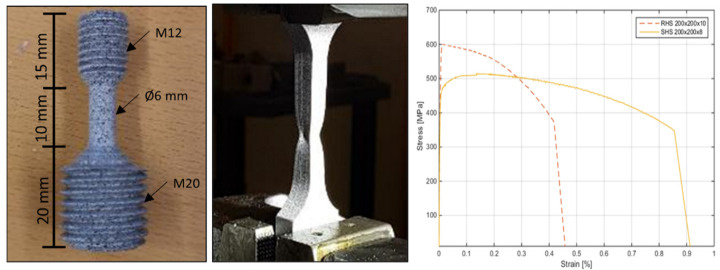
Cylindrical (**left**) and flat (**center**) test specimens for studs and tubes, and stress-strain curves of tubes (**right**).

In order to compare with some of the results obtained from the specimens by the Small Punch Tests (SPT), also tensile tests were carried out on the studs and on coupons extracted from the tubes. The treaded studs were machined to obtain a cylindrical dog-bone tensile specimen, whereas the specimens from the tube walls were mechanized as a flat test specimen (Figure 10) according to the ISO standard (EN ISO 6892-1). These specimens were examined in typical tensile test until failure in a universal testing machine MTS810 with a 100-kN load cell. In both cases, a Digital Image Correlation (DIC) system ARAMIS 5M with two 50-mm focal length cameras was used to obtain the strain in the surface of the specimen. The plotted curve load vs. strain was used to obtain the values of yield stress and tensile strength. As an example of these curves, the ones obtained for the tubes are presented in Figure 10 (right). 

Table 5 shows these data together with the ratio *f_u_/f_y_*, where the studs were coded by letter “S” followed by the diameter of the stud, class, length, and number of the specimen. The coupons from the RHS columns were coded with letter “C” followed by width, length, and thickness.

Steel rod, bars, and wire for cold heading and cold extrusion made of 8MnSi7 should present an *f_u_* higher than 520 MPa (as hot rolled or peeled) or more than 800 MPa if cold drawn [27], whereas class 4.8 should have a *f_u_* higher than 420 MPa [26]. In all cases, except one (779.16 MPa), the samples showed a higher ultimate tensile strength than the required one. The average yield strength was 654.2 and 482.0 MPa for K800-class and 4.8-class specimens, respectively, while the average ultimate tensile strength was 813.1 and 565.3 MPa for K800-class and 4.8-class specimens, respectively. These results mean deviations of 13.73 and 0.20%, respectively, with the mechanical properties of yield stress and ultimate tensile strength given by the manufacturer for K800-class studs. In 4.8-class studs, deviations of 11.82 and 13.14% were found between the results obtained by the authors and the mechanicals properties of yield stress and ultimate tensile strength given by the manufacturer, respectively.

With respect to the steel tubes, the obtained results on tensile tests with a DIC system were similar to the results given by the manufacturer *f_y_* = 426.85 MPa and *f_u_* = 541.01 MPa (SHS 200 × 200 × 8) and *f_y_* = 529.67 MPa and *f_u_* = 587.14 MPa (RHS 200 × 150 × 10).

## 3. Results

**Figure 11:**
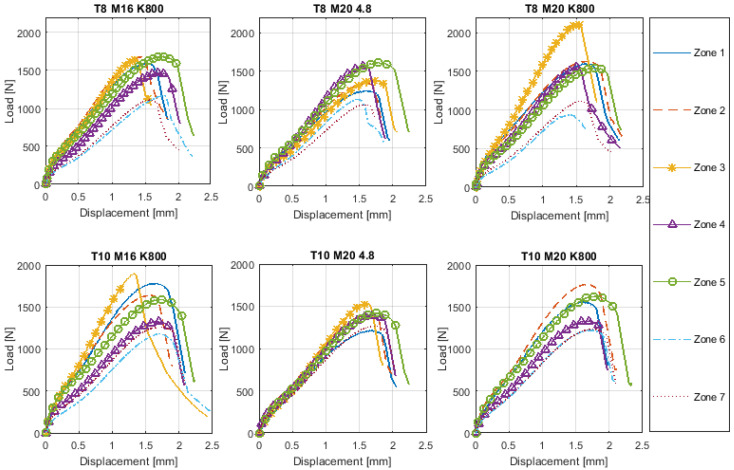
Load vs. displacement Small Punch Test (SPT) curves.

Figure 11 shows load versus displacement curves obtained from the SPT in the seven analyzed zones described in Figure 4 from each specimen.

Differences in behavior among analyzed zones were found. Although the results of Figure 11 were affected by the thickness of the specimen, some trends can be extracted. From the results it was inferred that zone 3, which corresponded to the interface between the stud and the tube, reached a higher maximum load with a lower maximum displacement than zone 2 and zone 4. This effect was clear in K800 studs, although not clear at all for 4.8 studs. Furthermore, stud class 4.8 showed a similar maximum value of loading but a different maximum value of displacement in zones 4 and 5. This phenomenon was not observed in K800 studs, where a lower force for zone 4 was obtained, except for T8 M20 K800 in which the loading for both zones was similar. It was also observed that the maximum value of loading reached was lower for zone 6 and 7 than for the rest of the analyzed areas. The reason for this phenomenon was because zones 6 and 7 were located on the wall of the tube (Figure 4) and the tubes presented lower mechanical properties than the studs. Comparing specimens from tube wall thicknesses 8 and 10 mm, different maximum load values were obtained for zones 6 and 7, with those obtained for a thickness of 10 mm being higher than in a case of a thickness of 8 mm.

The yield stress ($f_{y}$), ultimate tensile strength ($f_{u})$, and elongation (A) were estimated using Equations (4)–(6) respectively. These data are shown in Table 6, Table 7, Table 8, Table 9, Table 10 and Table 11. Due to a problem obtaining the specimen corresponding to zone 3 of T10 M20 K800 tube, this specimen could not be tested.

**Figure 12:**
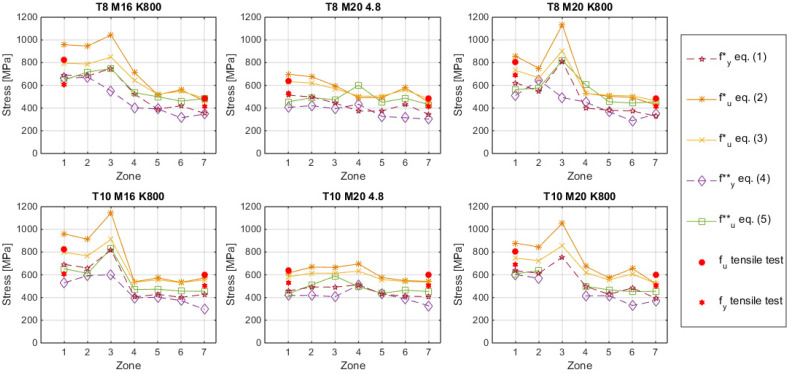
Yield stress and ultimate tensile strength profiles along different zones of the specimens.

A profile of the yield stress and ultimate tensile strength profiles captured in all approaches along the six combinations tube/stud considered in this research is shown in Figure 12. As can be seen in the figure, the highest mechanical properties were found in the adjacent areas of the welding bead (zones 2 or 3), while the lowest ones were found in zones 5, 6, and 7 (structural steel tubes).

## 4. Discussion

**Figure 13:**
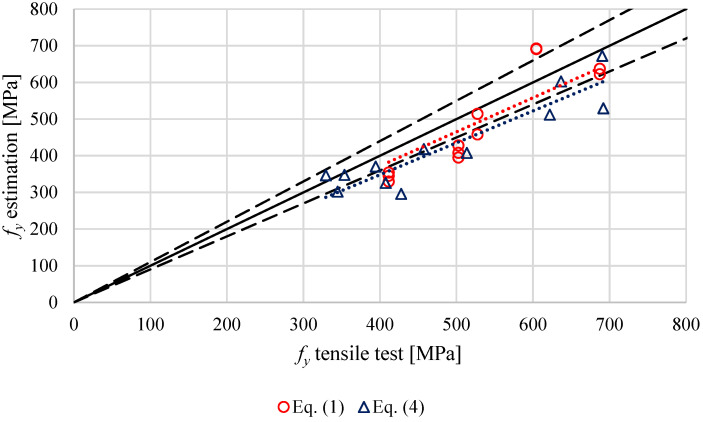
Comparison of yield stress values determined in the tensile tests and SPT (blue) or HV (red) estimations. Linear trendlines as dotted lines.

**Figure 14:**
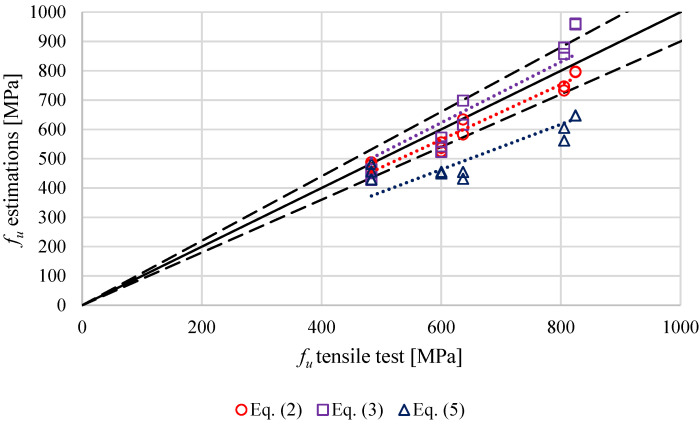
Comparison of ultimate tensile strength values determined in the tensile tests and SPT (blue) or HV (red) estimations. Linear trendlines as dotted lines.

The results obtained with the SPT and the estimated in zones 1 (stud, Figure 4) and zone 7 (plate, Figure 4) through the Equations (1)–(3) proposed by Fujita and Kuki [10] and Murphy and Arbtin [11] were compared with those obtained in the tensile tests. The yield stress obtained in the SPT through Equation (4) and the one estimated with Equation (1) proposed by Fujita are shown in Figure 13, while the comparison between the ultimate tensile strength obtained by the SPT trough Equation (5) and the estimated with the Equations (2) and (3), proposed by Murphy and Arbtin and Fujita and Kuki, were compared with the ultimate tensile strength obtained in the tensile test. Figure 14 shows these data. In both cases the dashed lines show a ±10% of deviation and the dotted lines the trends.

The better agreement between the results from estimations and the tensile tests was found using the ultimate tensile strength equations proposed by Fujita and Kuki, with an average absolute deviation of 5.7% and a maximum deviation of 12.6%. The ultimate tensile strength equations proposed by Murphy and Arbtin reached an average absolute deviation of 9.3% and a maximum deviation of 16.5%. The differences between the predictions and the tensile tests were a bit higher when the yield stress was estimated, with an average absolute deviation of 13.9% and a maximum deviation of 21.6%. In the case of the SPT estimations, the deviations reached an average absolute deviation of 20.8% with a maximum deviation of 32.1% for the ultimate tensile strength and an average absolute deviation of 20.3% with a maximum error of 35.2% for the yield stress. Comparing 4.8 and K800 stud classes, a slightly better agreement was found in 4.8 class studs, using HV estimations.

The estimated elongations through SPT revealed that stud class K800 presented a drop of elongation value in zone 3. This fact was in accordance with the observed increase in hardness, but in class 4.8 samples this fact was not noticed. Furthermore, K800-class studs increased yield strength and ultimate tensile strength in zone 3.

## 5. Conclusions

The results of a study of 4.8 and K800-class threaded studs with metrics M16 and M20 welded to RHS columns are presented in this work.

An analysis of the stud welding process with a thermal camera showed that high temperatures were located nearby the ceramic ferrule. This fact agreed with the small HAZ and small welding bead observed in the joint.

Hardness measurements were taken on seven different places along the studs/tube specimens in order to estimate the mechanical properties along the welded joint. The results showed that K800 class specimens had a higher hardness in zone C.

The visual study of the welding showed that porosity can appear in the welding bead. As porosity was associated with cracks onset [33] when the joint is subjected to dynamic loading, a shorter fatigue life can be expected [34]. Furthermore, a welding bead raised on one side can appear for the bigger the diameter of the stud because of the irregular current distribution in the work piece. For this reason, the authors suggested that non-destructive tests are recommended in order to verify the suitability of the welded joint; especially in K800-class studs, where cracks were found.

SPT was applied in seven zones along the welded stud. SPT showed differences in the maximum loading and in the displacement reached in the different zones. These values together with the equations proposed by [12] allowed us to estimate the mechanical properties in the different zones of the welded studs.

The estimation of the yield stress and ultimate tensile strength was made in two different ways: through SPT and through a correlation with the Vickers hardness number. When it was possible, tensile tests were performed in order to compare them with the estimations. The estimated elongations through SPT revealed that class K800 studs had a lower elongation in zone 3 than in zones 2 and 4.

The assessment of the indirect methods for the yield stress through Vickers hardness and SPT was made by comparing their results with the standard tensile tests in all cases in which it was possible. The estimation through the Vickers hardness proposed by Fujita and Kuki gave better agreement with the measured yield stresses than the proposal that uses the SPT results, being the deviation of this on the safe side.

Regarding the ultimate tensile strength, the two estimations through Vickers hardness also showed better agreement with the values directly obtained from the existing tensile tests than the SPT estimation, that is too conservative. Fujita and Kuki’s proposal matched better, but was slightly unconservative compared with Murphy and Arbtin’s one.

According to the previous points and the much easier procedure to obtain the Vickers hardness, it is preferable to use the material properties indirectly obtained from HV until further studies and calibrations on the SPT estimations of material properties are carried out on these specific steels.

## Figures and Tables

**Table 1:** Combinations of tube thicknesses, stud classes (K800 and 4.8), and stud diameters (16 mm and 20 mm) tested.

Tube Thicknesses	Stud Class	M16	M20
8 mm	K800	X	X
	4.8		X
10 mm	K800	X	X
	4.8		X

**Table 2:** Limitations on chemical composition (%) of the 4.8 steel grade, 8MnSi7, and S355J2H.

Steel	$C_{\max}$	Si	Mn	$P_{\max}$	$S_{\max}$	Al
4.8	0.55	-	-	0.05	0.06	-
8MnSi7 (1.5113)	0.10	0.90 to 1.10	1.60 to 1.80	0.025	0.025	≤0.020
S355J2H (1.0577)	0.22	≤0.55	≤1.60	0.035	0.035	>0.02

**Table 3:** Chemical composition of the tubes and studs. Composition in %.

Steel	C	Si	Mn	P	S	Al
S355 J2H (SHS 200 × 200 × 8)	0.17	0.011	1.191	0.011	0.005	0.027
S355 J2H (RHS 200 × 150 × 10)	0.070	0.014	0.850	0.013	0.005	0.025
8MnSi7 (M20)	0.094	0.994	1.67	0.015	0.003	0.011
8MnSi7 (M16)	0.089	1	1.67	0.013	0.005	0.012
4.8	0.14	-	0.4	0.012	0.009	0.03

**Table 4:** Vickers hardness in the different zones of the tubes.

Tube Code	Vickers Hardness Number						
	Zone B	Zone C	Zone D	Zone E	Zone F	Zone G	Zone H
T8 M16 K800	278.14	275.13	299.37	217.23	168.48	179.37	155.03
T8 M20 4.8	213.50	207.88	188.17	162.07	162.42	183.82	151.76
T8 M20 K800	253.1	226.10	321.6	171.67	164.93	162.67	146.10
T10 M16 K800	278.70	266.87	324.66	173.77	182.19	172.51	182.08
T10 M20 4.8	192.98	205.93	205.55	212.76	183.16	176.30	174.68
T10 M20 K800	258.48	249.98	302.07	207.87	182.21	202.94	169.91

**Table 5:** Mechanical properties of materials of columns and studs captured in tensile tests.

Specimen	*f_y_* [MPa]	*f_u_* [MPa]	*f_u_/f_y_*
S16-4.8-30-1	455.2	493.7	1.08
S16-4.8-30-2	417.5	494.2	1.18
S16-K800-40-1	579.7	834.5	1.44
S16-K800-40-2	629.1	814.5	1.29
S20-4.8-35-1	573.5	638.4	1.11
S20-4.8-35-2	481.7	634.9	1.32
S20-K800-50-1	642.6	800.9	1.25
S20-K800-50-2	735.7	836.6	1.14
S20-K800-50-3	683.7	779.2	1.14
C200×150×10	502.7	600.3	1.19
C200 × 200 × 8	411.5	483.4	1.17

**Table 6:** Thickness, SPT results, and estimated properties through HV (*) and SPT (**) of the T8 M16 K800 specimen.

Zone	$\bar{t}$ [mm]	HV			SPT					
		$f_{y}^{*}$ Equation (1) [MPa]	$f_{u}^{*}$ Equation (2) [MPa]	$f_{u}^{*}$ Equation (3) [MPa]	*d* [mm]	$P_{max}$/t·d [MPa]	$P_{y}$/t^2^ [MPa]	$f_{y}^{**}$ Equation (4) [MPa]	$f_{u}^{**}$ Equation (5) [MPa]	A^**^ Equation (6) [%]
1	0.444	690.5	958.1	795.3	1.54	2336.4	1943.2	672.4	647.2	21.02
2	0.447	682.3	946.0	787.8	1.36	2776.1	1949.2	674.4	769.0	18.43
3	0.453	748.6	1043.4	848.4	1.35	2694.6	1660.5	574.5	746.4	18.10
4	0.443	523.8	713.2	643.1	1.72	1927.6	1162.2	402.1	533.9	23.51
5	0.534	390.5	517.3	521.2	1.75	1803.5	1133.8	392.3	499.6	19.84
6	0.394	420.3	561.1	548.4	1.78	1666.7	910.8	315.1	461.7	27.43
7	0.410	353.7	463.2	487.6	1.56	1742.4	1004.2	347.5	482.7	23.13

**Table 7:** Thickness, SPT results, and estimated properties through HV (*) and SPT (**) of the T8 M20 4.8 specimen.

Zone	$\bar{t}$ [mm]	HV			SPT					
		$f_{y}^{*}$ Equation (1) [MPa]	$f_{u}^{*}$ Equation (2) [MPa]	$f_{u}^{*}$ Equation (3) [MPa]	*d* [mm]	$P_{max}$/t·d [MPa]	$P_{y}$/t^2^ [MPa]	$f_{y}^{**}$ Equation (4) [MPa]	$f_{u}^{**}$ Equation (5) [MPa]	A^**^ Equation (6) [%]
1	0.472	513.6	698.2	633.7	1.60	1642.1	1177.3	407.3	454.9	20.61
2	0.471	498.3	675.6	619.7	1.60	1774.2	1215.4	420.5	491.5	20.64
3	0.468	444.3	596.4	570.4	1.71	1729.5	1138.4	393.9	479.1	22.10
4	0.462	372.9	491.5	505.2	1.56	2171.5	1121.6	388.1	601.5	20.49
5	0.553	373.9	492.9	506.1	1.78	1631.8	941.6	325.8	452.0	19.54
6	0.435	432.4	578.9	559.6	1.49	1743.0	911.1	315.2	482.8	20.82
7	0.430	344.7	450.1	479.4	1.59	1550.6	872.6	301.9	429.5	22.43

**Table 8:** Thickness, SPT results, and estimated properties through HV (*) and SPT (**) of the T8 M20 K800 specimen.

Zone	$\bar{t}$ [mm]	HV			SPT					
		$f_{y}^{*}$ Equation (1) [MPa]	$f_{u}^{*}$ Equation (2) [MPa]	$f_{u}^{*}$ Equation (3) [MPa]	*d* [mm]	$P_{max}$/t·d [MPa]	$P_{y}$/t^2^ [MPa]	$f_{y}^{**}$ Equation (4) [MPa]	$f_{u}^{**}$ Equation (5) [MPa]	A^**^ Equation (6) [%]
1	0.479	622.0	857.4	732.8	1.64	2028.6	1479.0	511.7	561.9	20.86
2	0.476	548.1	748.9	665.3	1.64	2083.9	1874.4	648.5	577.2	20.99
3	0.471	809.4	1132.8	904.0	1.52	2960.4	1719.0	594.8	820.0	19.57
4	0.469	399.2	530.1	529.2	1.52	2185.5	1310.9	453.6	605.4	19.64
5	0.527	380.8	503.0	512.3	1.77	1646.2	1057.6	365.9	456.0	20.42
6	0.418	374.6	493.9	506.7	1.40	1602.3	835.8	289.2	443.8	20.39
7	0.429	329.2	427.3	465.3	1.65	1648.6	1001.2	346.4	456.7	23.28

**Table 9:** Thickness, SPT results, and estimated properties through HV (*) and SPT (**) of the T10 M16 K800 specimen.

Zone	$\bar{t}$ [mm]	HV			SPT					
		$f_{y}^{*}$ Equation (1) [MPa]	$f_{u}^{*}$ Equation (2) [MPa]	$f_{u}^{*}$ Equation (3) [MPa]	*d* [mm]	$P_{max}$/t·d [MPa]	$P_{y}$/t^2^ [MPa]	$f_{y}^{**}$ Equation (4) [MPa]	$f_{u}^{**}$ Equation (5) [MPa]	A^**^ Equation (6) [%]
1	0.465	692.0	960.3	796.7	1.64	2338.6	1529.7	529.3	647.8	21.36
2	0.471	659.7	912.8	767.2	1.57	2212.9	1717.7	594.3	612.9	20.29
3	0.474	817.8	1145.1	911.6	1.34	2993.3	1736.2	600.7	829.1	17.15
4	0.467	404.9	538.5	534.4	1.67	1700.5	1148.6	397.4	471.0	21.67
5	0.540	428.0	572.4	555.5	1.71	1713.8	1161.1	401.8	474.7	19.24
6	0.420	401.5	533.5	531.3	1.71	1646.2	1080.3	373.8	456.0	24.70
7	0.442	427.7	571.9	555.2	1.74	1642.2	854.1	295.5	454.9	23.83

**Table 10:** Thickness, SPT results, and estimated properties through HV (*) and SPT (**) of the T10 M20 4.8 specimen.

Zone	$\bar{t}$ [mm]	HV			SPT					
		$f_{y}^{*}$ Equation (1) [MPa]	$f_{u}^{*}$ Equation (2) [MPa]	$f_{u}^{*}$ Equation (3) [MPa]	*d* [mm]	$P_{max}$/t·d [MPa]	$P_{y}$/t^2^ [MPa]	$f_{y}^{**}$ Equation (4) [MPa]	$f_{u}^{**}$ Equation (5) [MPa]	A^**^ Equation (6) [%]
1	0.465	457.5	615.8	582.5	1.68	1558.1	1208.0	418.0	431.6	22.09
2	0.471	492.9	667.8	614.8	1.64	1847.9	1212.9	419.7	511.9	21.51
3	0.474	491.9	666.3	613.9	1.61	2125.9	1174.9	406.5	588.9	21.76
4	0.467	511.6	695.3	631.9	1.71	1762.6	1471.5	509.1	488.2	22.35
5	0.540	430.6	576.3	557.9	1.76	1578.5	1249.6	432.4	437.2	20.79
6	0.420	411.9	548.7	540.8	1.00	1675.7	1118.5	387.0	464.2	14.18
7	0.442	407.4	542.2	536.7	1.72	1643.6	941.5	325.7	455.3	23.34

**Table 11:** Thickness, SPT results, and estimated properties through HV (*) and SPT (**) of the T10 M20 K800 specimen.

Zone	$\bar{t}$ [mm]	HV			SPT					
		$f_{y}^{*}$ Equation (1) [MPa]	$f_{u}^{*}$ Equation (2) [MPa]	$f_{u}^{*}$ Equation (3) [MPa]	*d* [mm]	$P_{max}$/t·d [MPa]	$P_{y}$/t^2^ [MPa]	$f_{y}^{**}$ Equation (4) [MPa]	$f_{u}^{**}$ Equation (5) [MPa]	A^**^ Equation (6) [%]
1	0.441	636.7	879.0	746.2	1.62	2186.3	1741.6	602.6	605.6	22.23
2	0.460	613.5	844.9	725.0	1.68	2293.1	1642.9	568.4	635.2	22.12
3	0.451	756.0	1054.3	855.2	-	-	-	-	-	-
4	0.445	498.2	675.6	619.7	1.67	1793.2	1195.8	413.7	496.7	22.76
5	0.535	428.0	572.5	555.5	1.80	1687.9	1204.0	416.6	467.6	20.44
6	0.434	484.7	655.8	607.3	1.73	1625.5	952.0	329.4	450.3	24.18
7	0.433	394.4	523.0	524.8	1.76	1623.7	1069.9	370.2	449.8	24.61

## Data Availability

The data presented in this study are available within the article. Any other data that the reader could considered necessary to reproduce the experimental work are available on request from the corresponding author.

## References

[1] Wang Y., Xue L. (2013). Experimental study of moment–rotation characteristics of reverse channel connections to tubular columns. J. Constr. Steel Res..

[2] Elghazouli A., Málaga-Chuquitaype C., Castro J.M., Orton A. (2009). Experimental monotonic and cyclic behavior of blind-bolted angle connections. Eng. Struct..

[3] Thai H.-T., Uy B. (2016). Rotational stiffness and moment resistance of bolted endplate joints with hollow or CFST columns. J. Constr. Steel Res..

[4] Thai H.-T., Uy B., Yamesri, Aslani F. (2017). Behaviour of bolted endplate composite joints to square and circular CFST columns. J. Constr. Steel Res..

[5] Maquoi R., Naveau X., Rondal J. (1984). Beam-column welded stud connections. J. Constr. Steel Res..

[6] Vandegans D. (1996). Use of Threaded Studs in Joints between I-Beam and RHS-Column. IABSE Rep..

[7] ISO 6892-1:2016 (2016). Metallic Materials—Tensile Testing—Part 1: Method of Test at Room Temperature.

[8] Kameda J., Mao X. (1992). Small-punch and TEM-disc testing techniques and their application to characterization of radiation damage. J. Mater. Sci..

[9] Rodríguez C., García Cabezas J., Cárdenas E., Belzunce F.J., Betegón C. (2009). Mechanical Properties Characterization of Heat-Affected Zone Using the Small Punch Test. Weld. J..

[10] Fujita M., Kuki K. (2016). An Evaluation of Mechanical Properties with the Hardness of Building Steel Structural Members for Reuse by NDT. Metals.

[11] Murphy G., Arbtin E. (1953). Correlation of Vickers Hardness Number, Modulus of Elasticity, and the Yield Strength for Ductile Metals.

[12] García T., Rodríguez C., Belzunce F., Suárez C. (2014). Estimation of the mechanical properties of metallic materials by means of the small punch test. J. Alloys Compd..

[13] Manahan M., Argon A., Harling O. (1981). The development of a miniaturized disk bend test for the determination of postirradiation mechanical properties. J. Nucl. Mater..

[14] Huang F.H., Hamilton M.L., Wire G.L. (1982). Bend Testing for Miniature Disks. Nucl. Technol..

[15] Baik J.-M., Kameda J., Buck O. (2008). Development of Small Punch Tests for Ductile-Brittle Transition Temperature Measurement of Temper Embrittled Ni-Cr Steels. The Use of Small-Scale Specimens for Testing Irradiated Material.

[16] Foulds J., Viswanathan R. (1994). Small Punch Testing for Determining the Material Toughness of Low Alloy Steel Components in Service. J. Eng. Mater. Technol..

[17] Ha J.S., Fleury E. (1998). Small punch tests on steels for steam power plant(I). KSME Int. J..

[18] Altstadt E., Houska M., Simonovski I., Bruchhausen M., Holmström S., LaCalle R. (2018). On the estimation of ultimate tensile stress from small punch testing. Int. J. Mech. Sci..

[19] Cuesta I., Alegre J. (2011). Determination of the fracture toughness by applying a structural integrity approach to pre-cracked Small Punch Test specimens. Eng. Fract. Mech..

[20] Guan K., Hua L., Wang Q., Zou X., Song M. (2011). Assessment of toughness in long term service CrMo low alloy steel by fracture toughness and small punch test. Nucl. Eng. Des..

[21] Konopík P., Džugan J., Procházka R. Determination of Fracture Toughness and Tensile Properties of Structural Steels by Small Punch Test and Micro-Tensile Test. Proceedings of the METAL 2013—22nd International Conference on Metallurgy and Materials, Conference Proceedings.

[22] Rodriguez C.F., Cárdenas E., Belzunce F.J., Betegón C. (2013). Fracture Characterization of Steels by Means of the Small Punch Test. Exp. Mech..

[23] CEN Workshop Agreement CWA 15627:2006 (2006). Small Punch Test Method for Metallic Materials.

[24] Bruchhausen M., Austin T., Holmström S., Altstadt E., Dymacek P., Jeffs S., Lancaster R., LaCalle R., Matocha K., Petzová J. (2017). European Standard on Small Punch Testing of Metallic Materials. American Society of Mechanical Engineers, Pressure Vessels and Piping Division (Publication) PVP.

[25] EN ISO14555 (2017). Welding. Arc Stud Welding of Metallic Materials.

[26] EN ISO 898-1 (2015). Mechanical Properties of Fasteners Made of Carbon Steel and Alloy Steel. Part 1: Bolts, Screws and Studs with Specified Property Classes. Coarse Thread and Fine Pitch Thread.

[27] EN 10263-2 (2018). Steel Rod, Bars and Wire for Cold Heading and Cold Extrusion. Part 2: Technical Delivery Conditions for Steels Not Intended for Heat Treatment after Cold Working.

[28] EN 10219-1 (2007). Cold Formed Welded Steel Structural Hollow Sections of Non-Alloy and Fine Grain Steels Part 1: Technical Delivery Conditions.

[29] Ginzburg V.B., Ballas R. (2000). Flat Rolling Fundamentals.

[30] EN ISO 15614-1 (2018). Specification and Qualification of Welding Procedures for Metallic Materials. Welding Procedure Test. Part 1: Arc and Gas Welding of Steels and Arc Welding of Nickel and Nickel Alloys.

[31] Taylor M., Choi K., Sun X., Matlock D., Packard C., Xu L., Barlat F. (2014). Correlations between nanoindentation hardness and macroscopic mechanical properties in DP980 steels. Mater. Sci. Eng. A.

[32] Hashemi S. (2011). Strength–hardness statistical correlation in API X65 steel. Mater. Sci. Eng. A.

[33] Holmes J., Queeney R.A. (1985). Fatigue Crack Initiation in a Porous Steel. Powder Met..

[34] Lawrence F.V., Ho N.J., Mazumdar P.K. (1981). Predicting the Fatigue Resistance of Welds. Annu. Rev. Mater. Res..

